# Optical Memory in a MoSe_2_/Clinochlore Device

**DOI:** 10.1021/acsami.4c19337

**Published:** 2025-02-13

**Authors:** Alessandra Ames, Frederico B. Sousa, Gabriel A. D. Souza, Raphaela de Oliveira, Igor R. F. Silva, Gabriel L. Rodrigues, Kenji Watanabe, Takashi Taniguchi, Gilmar E. Marques, Ingrid D. Barcelos, Alisson R. Cadore, Victor Lopez-Richard, Marcio D. Teodoro

**Affiliations:** † Departamento de Física, Universidade Federal de São Carlos, São Carlos, São Paulo 13565-905, Brazil; ‡ Brazilian Synchrotron Light Laboratory (LNLS), Brazilian Center for Research in Energy and Materials (CNPEM), Campinas, São Paulo 13083-100, Brazil; § Brazilian Nanotechnology National Laboratory (LNNano), Brazilian Center for Research in Energy and Materials (CNPEM), Campinas, São Paulo 13083-200, Brazil; ∥ “Gleb Wataghin” Institute of Physics, State University of Campinas, Campinas, São Paulo 13083-970, Brazil; ⊥ Research Center for Electronic and Optical Materials, National Institute for Materials Science, 1-1 Namiki, Tsukuba 305-0044, Japan; # Research Center for Materials Nanoarchitectonics, National Institute for Materials Science, 1-1 Namiki, Tsukuba 305-0044, Japan; ¶ Programa de Pós-Graduação em Física, Instituto de Física, Universidade Federal do Mato Grosso, Cuiabá 79070-900, Brazil

**Keywords:** phyllosilicates, 2D natural materials, optical memory effect, MoSe_2_/clinochlore, charge dynamics

## Abstract

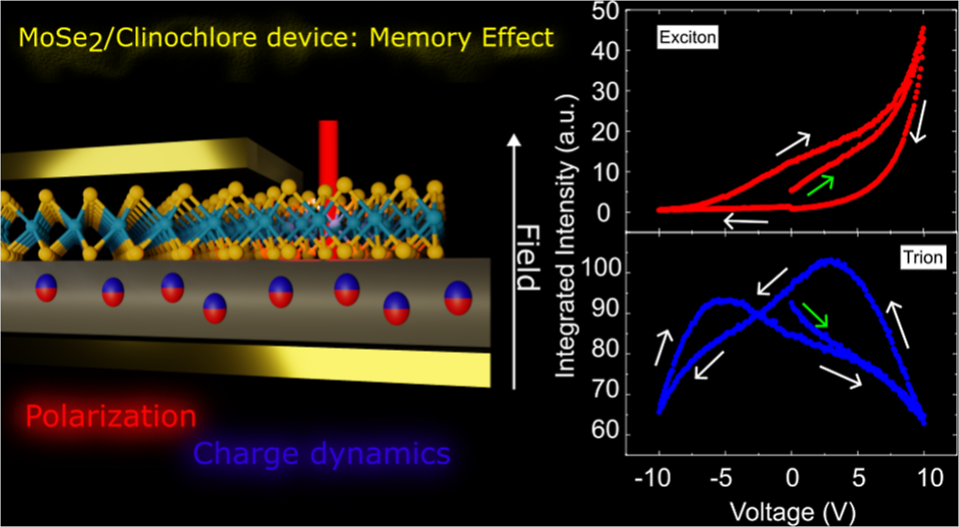

Two-dimensional heterostructures have been crucial in advancing optoelectronic devices utilizing van der Waals materials. Semiconducting transition-metal dichalcogenide monolayers, known for their unique optical properties, offer extensive possibilities for light-emitting devices. Recently, a memory-driven optical device, termed a Mem-emitter, was proposed by using these monolayers atop dielectric substrates. The successful realization of such devices heavily depends on the selection of the optimal substrate. Here, we report a pronounced memory effect in a MoSe_2_/clinochlore device, evidenced by an electric hysteresis in the intensity and energy of MoSe_2_ monolayer emissions. This demonstrates both population- and transition-rate-driven Mem-emitter abilities. Our theoretical approach correlates these memory effects with internal state variables of the substrate, emphasizing that a clinochlore-layered structure is crucial for a robust and rich memory response. This work introduces a novel two-dimensional device with promising applications in memory functionalities, highlighting the importance of alternate insulators in the fabrication of van der Waals heterostructures.

## 1 Introduction

The confinement of electrons in two dimensions has led to novel physical phenomena extensively studied over the past few years in isolated atomically thin layers of van der Waals (vdW) materials.^1−5^ Among these phenomena, memory effects, such as resistive memory responses, in two-dimensional (2D) materials have garnered significant interest due to their potential computing functionalities.^6−10^ Recently, a theoretical proposal introduced an emitting device with memory capabilities based on its exposure history to light or other stimuli, introducing a new class of devices termed Mem-emitters.^11^

Semiconducting 2D transition-metal dichalcogenides (TMDs) are promising candidates for the active medium of Mem-emitter devices due to their unique optical and electronic properties.^2−5,12^ TMD monolayers exhibit a high carrier mobility,^13^ a direct band gap in the visible range,^14−16^ strong many-body effects,^17−22^ and tunable properties through doping,^23−25^ strain,^26−31^ and electric fields,^16,32−35^ making them ideal for Mem-emitter applications.^11^

Additionally, the performance of the TMD-based devices is strongly dependent on the interface between the active material and the insulator that separates it from the gate electrode.^16,30,36−38^ Thus, significant efforts are being made to identify suitable insulating 2D materials that preserve the qualities of the 2D semiconductor in vdW heterostructures (vdWHS).^36−38^ Hexagonal boron nitride (hBN), the most extensively studied vdW insulator, is chemically inert and has a flat surface free of dangling bonds,^36,37,39^ making it widely used as a gate insulator. Despite its advantages, the high cost of 2D hBN poses an obstacle to its use in large-scale nanodevice fabrication, highlighting the need for alternative vdW insulators.^38,40^

Phyllosilicates are naturally abundant vdW insulators that have recently emerged as promising materials for use in vdWHS.^33,38,40−48^ Among these phyllosilicates, clinochlore is a layered crystal with the general chemical formula Mg_5_Al(Si_3_Al)O_10_(OH)_8_, which, similarly to hBN, exhibits a wide band gap,^49^ a flat surface over large areas,^49^ and a dielectric constant of ∼4.3.^50^ However, clinochlore is a hydrated mineral^51,52^ that naturally contains impurities, point defects, and water nanoconfined in the vdW gap that might affect its insulating properties and interactions in a vdWHS.^49,51,52^ In contrast to hBN, the properties of clinochlore when integrated with other layered materials still require a thorough investigation.

Here, we report a robust optical memory effect in a vdWHS device based on monolayer (1L) MoSe_2_ atop ultrathin clinochlore flakes. We conducted photoluminescence (PL) spectroscopy experiments to examine the 1L-MoSe_2_ light emission under an external voltage bias. Optical memory traces in PL responses have been reported by the refs (53 and 54) and subsequently analyzed in detail.^55,56^ In the present research, hysteresis in the intensity and energy of both exciton and trion emissions emerges when the gate voltage is swept at an appropriate rate. This effect can also be tuned by varying the sweep amplitude and excitation power. Notably, the negligible hysteresis loop in reference 1L-MoSe_2_/hBN devices suggests that clinochlore layers play a crucial role in this optical memory effect. Calculations based on voltage-dependent polarization fluctuations and charge carrier population processes allowed us to correlate the experimentally observed hysteresis with dynamic internal state variables. We experimentally confirm that excitons in 1L-TMDs act as naturally transition-rate-driven Mem-emitters, while trions exhibit a combination of both transition-rate and population-driven abilities, as reported in ref (11). The theoretical outcomes indicate that disordered insulators are more suitable for enabling optical memory effects, corroborating the rich memory response observed in our clinochlore-based device. Thus, our results demonstrate the experimental realization of a Mem-emitter device based on vdWHS of a 1L-TMD on clinochlore layers, highlighting the potential of phyllosilicates as gate insulators in future nanotechnological devices.

## 2 Results and Discussion

**Figure 1:**
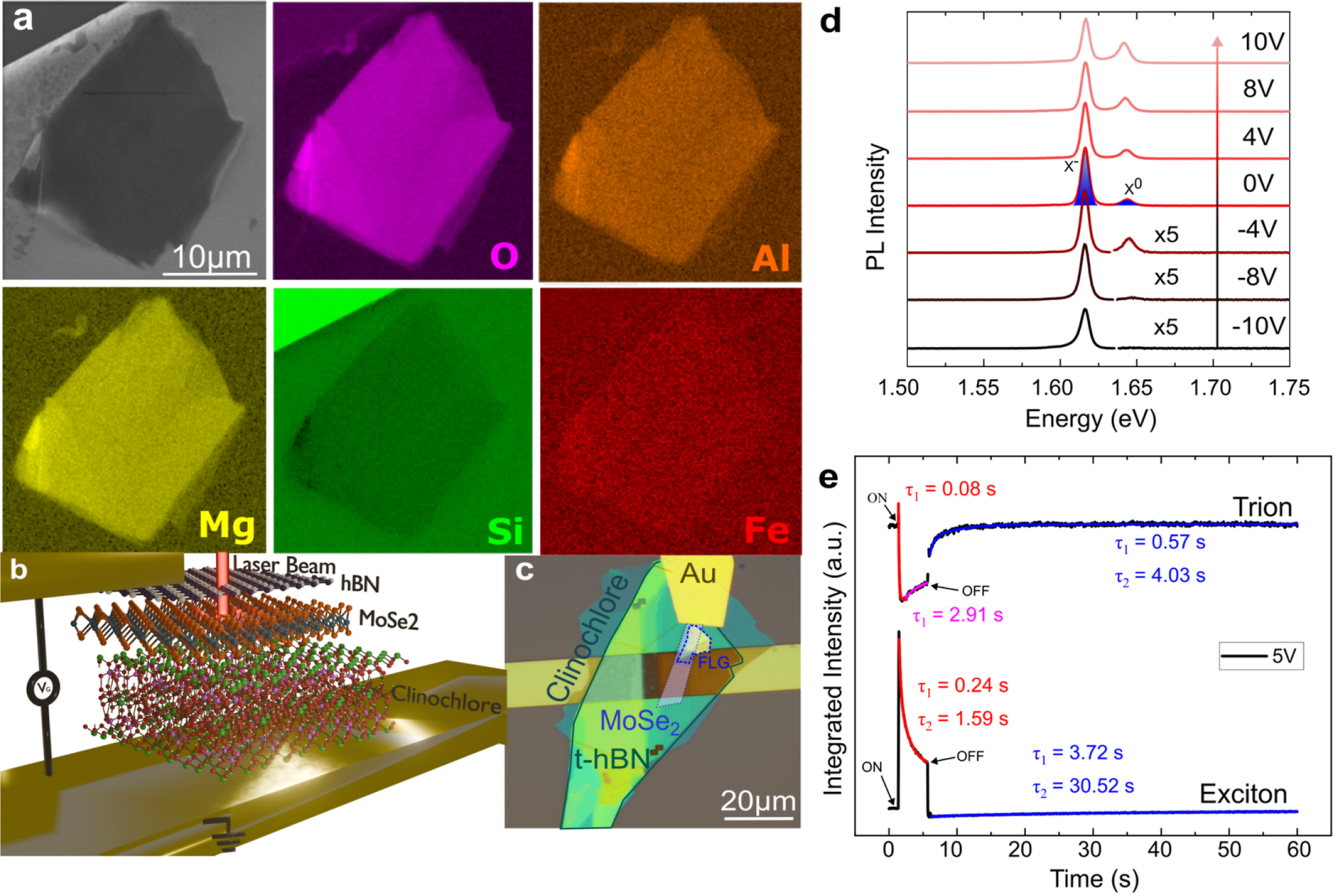
Clinochlore-based MoSe_2_ device. (a) Clinochlore SEM image and EDS chemical maps of its constituent elements in addition to Fe impurity. (b,c) Schematic view; (b) and optical microscopy image (c) of our *t*-hBN/1L-MoSe_2_/clinochlore/Au-electrode device. (d) PL spectra of 1L-MoSe_2_ acquired in the clinochlore sample at different gate voltages ranging from −10 to 10 V. X^0^ and X^–^ emissions are denoted in the PL spectrum at 0 V. (e) Time-resolved PL emission transients recorded for the integrated intensity of excitons and trions in the 1L-MoSe_2_ under the application of 5 s rectangular voltage pulses with a 5 V amplitude. Each spectrum was taken over 0.03 s.

The natural clinochlore crystal used in this study was precharacterized using several experimental techniques detailed elsewhere.^49−52^ Here, we exfoliated our natural sample onto a Si/Au (100 nm) substrate and performed hyperspectral mapping using energy-dispersive spectroscopy (EDS) to confirm the homogeneous composition of the obtained flakes before the sample fabrication process. The scanning electron microscopy (SEM) image of clinochlore with its elemental EDS maps is shown in Figure 1a. We observed a homogeneous distribution of the expected elements Mg, O, Si, and Al, along with Fe impurities typically present in natural clinochlore samples. The low chemical contrast for Fe impurities is related to their concentration (about 6 wt %^49^), which is close to the detection limit of the EDS resolution (approximately 2–5%). The low contrast in the Si map is due to the overlap with the Si content of the substrate.

After confirming the homogeneity of the exfoliated clinochlore sample composition, we proceeded with sample fabrication. Figure 1b presents a schematic view of our clinochlore-based device in a capacitor-like structure. 1L-MoSe_2_ is sandwiched between the top thin hBN and the bottom clinochlore flakes. Electrical contact from the Au electrode to 1L-MoSe_2_ was achieved through a few-layer graphite (FLG), with a voltage bias applied between the 1L-MoSe_2_ and the bottom Au electrode (grounded contact). See the Methods section and Section S1 for further details regarding sample fabrication and the insulating behavior of clinochlore (Figure S1), respectively. An optical microscope image of our fabricated hBN/FLG/1L-MoSe_2_/clinochlore/Au-electrode device, with each part highlighted, is shown in Figure 1c. Additionally, a similar sample with the bottom layer formed by hBN instead of clinochlore was fabricated as a reference device (Figure S2). Hereafter, the samples will be termed the clinochlore and reference samples.

Figure 1d shows the 1L-MoSe_2_ PL spectra obtained from the clinochlore sample at 3.6 K, with gate voltages ranging from −10 to 10 V. Emissions from neutral (X^0^) and charged (X^–^, trion) excitons are marked in the PL spectrum at 0 V, centered at 1.645 and 1.616 eV, with full widths at half-maximum (fwhm) of 8.9 and 6.7 meV, respectively. The corresponding PL spectra of the reference sample, shown in the Supporting Information (Figure S2), exhibit emission energies (fwhm) of 1.644 eV (4.3 meV) and 1.617 eV (4.0 meV) for X^0^ and X^–^ peaks at 0 V, respectively. These energy and fwhm values were extracted by fitting the spectra to two Lorentzian peaks, aligning with the values reported in the literature for hBN-encapsulated 1L-WSe_2_.^57^ These results suggest that the clinochlore is a viable alternative 2D insulator for emitting devices despite its heterogeneities.

Regarding the gate-dependent PL of the clinochlore sample, Figure 1d illustrates a red-shift and an increase in intensity for the X^0^ emission with increasing gate voltage. In contrast, the X^–^ emission shows a blue shift and a relative decrease in intensity with the voltage. Because the ground electrode is located at the bottom of the device, a positive gate voltage removes electrons from the 1L-MoSe_2_, while a negative gate voltage injects electrons. This evolution of the PL spectra highlights the negative character of the charged exciton, with the X^–^ emission dominating the spectra at negative voltages.^17,58−60^

**Figure 2:**
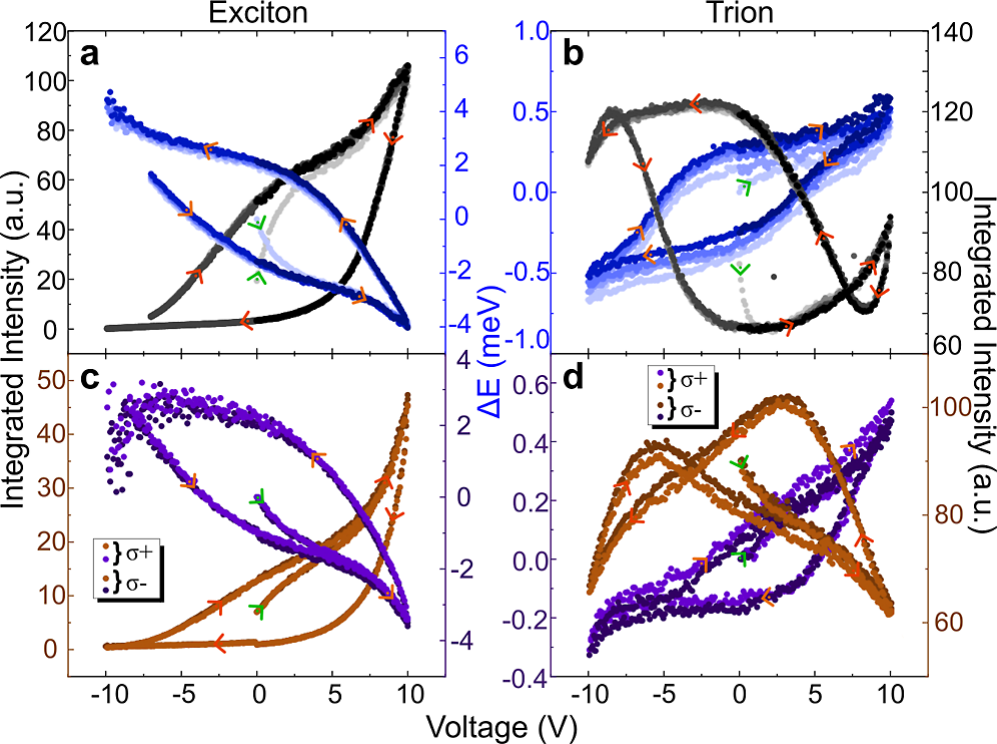
Hysteresis effect in the PL emission of the clinochlore device under external voltage sweeps of 18 min period. (a,b) Integrated intensity (in shades of black) and energy shift (in shades of blue) of X^0^ (a) and X^–^ (b) emissions as a function of the gate voltage, which was subsequently swept 5 times in cycles ranging from −10 to 10 V. Each voltage cycle is presented in a different shade of black or blue for the intensity or energy data, respectively. (c,d) Integrated intensity (in shades of brown) and energy shift (in shades of purple) of X^0^ (c) and X^–^ (d) emissions, after defect passivation, are shown as a function of the gate voltage. These measurements were detected at σ^+^ and σ^–^ circular polarizations. The voltage cycle for each detection is presented in a different shade of brown or purple for the intensity or energy data, respectively. For all graphs, green arrows indicate where the measurement initiates and orange arrows denote the direction of the voltage sweep. The energy shift Δ*E* is relative to the first emission energy measured at 0 V.

The minor blue shift of the trion emission, approximately 1 meV, is not readily apparent in the PL spectra of Figure 1d due to its magnitude being an order of magnitude smaller than the trion’s fwhm (∼6.7 meV). However, the hysteresis loops shown in Figure 2b clearly confirm this behavior, providing a more precise representation of the trion energy shift under varying gate voltages.

An external electric field can modify internal state variables, such as polarization or charge carrier populations. For an emitting device based on a 1L-TMD atop a dielectric substrate, these changes impact the optical properties of the TMD.^11^ The dynamics of these internal state variable modifications create the conditions necessary for observing memory effects.^11^ The most straightforward way to prove the existence of nonequilibrium processes along with their proper time scales is through the analysis of time-resolved transients of the observables under a constant bias.

To examine transient processes, we investigated the temporal dependence of the 1L-MoSe_2_ PL spectrum obtained from the clinochlore sample by continuously acquiring several PL spectra over 60 s, as shown in Figure 1e. This measurement began at 0 V, and after 1 s, a gate voltage of 5 V was applied, resulting in an abrupt decrease (increase) in the X^–^ (X^0^) emission intensity. At 5.5 s, the voltage was turned off again.

For our 1L-MoSe_2_ device, the optical properties are influenced by fluctuations in the substrate’s polarization and carrier population under an applied voltage. We have then correlated a recent theoretical model that outlines the electric hysteresis of optical emission intensity and energy for 2D devices^11^ with our experimental data.

The dynamics of the nonequilibrium carrier population are governed by independent relaxation mechanisms, each characterized by relaxation times τ_*i*_^(*n*)^.^11^ The evolution of the fluctuation δ*n*_*i*_ in the carrier population responds as^11^
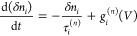
1where *n* = *n*_0_ + ∑_*i*_δ*n*_*i*_ represents the carrier population fluctuating around certain equilibrium values. Similarly, polarization fluctuations *P* = *P*_0_ + ∑_*j*_δ*P*_*j*_ follow an analogous dynamic, described by
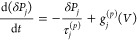
2with τ_*j*_^(*p*)^ denoting the relaxation times for polarization dynamics. Here, *g*_*i*_^(*n*)^(*V*) and *g*_*j*_^(*p*)^(*V*) are the nonequilibrium carrier and polarization transfer functions, respectively, both dependent on the external gate voltage, *V*.^61,62^ According to ref (11), the emission energies of both excitons and trions evolve proportionally to ε_0_*V*/*d*_2_ + ∑_*j*_δ*P*_*j*_(*t*), *d*_2_ being the thickness of the dielectric substrate. Similarly, the exciton intensity follows this relationship, while the trion intensity is proportional to the amount of the electrons, *n*_0_ + ∑_*i*_δ*n*_*i*_(*t*).^63^

The coexistence of dynamics with contrasting relaxation times was confirmed through exponential decay fits using the solutions of eqs 1 and 2,^11^ as displayed in Figure 1e. Hence, these memory phenomena are governed by processes spanning a wide temporal scale, from fractions of seconds to minutes.

To reveal the memory effects of the device, using the slowest dynamics as a reference, we obtained 1L-MoSe_2_ PL spectra from the clinochlore sample by sweeping the gate voltage (0 V → 10 V → −10 V → 0 V) with steps of 0.1 V and a cycling period *T* until stable closed cycles were achieved. From these spectra, we determined the intensity (in black) and energy (in blue) of X^0^ and X^–^ emissions as a function of the gate voltage, as shown in Figure 2a,b.

A Mem-emitter response emerges for both emissions in intensity and energy when sweeping the voltage. The hysteresis of the X^0^ emission, as displayed in Figure 2a, presents a leaf shape with no crossings and a clockwise (counterclockwise) loop direction for the intensity (energy). In contrast, the hysteresis of the X^–^ emission, in Figure 2b, exhibits a bow-like topology with two crossing points at opposite voltages for intensity concomitant with a leaf shape for energy and a counterclockwise (clockwise) loop direction for the intensity (energy) around *V* = 0 V.

Figure 2a,b demonstrates that the hysteresis loops are consistent across the five measured cycles. Their reproducibility is further confirmed in Figure S3, which shows similar hysteresis loops for an experiment conducted under the same conditions but with the gate voltage cycle starting in the opposite direction. Additionally, we performed the hysteresis measurements detecting emissions σ^+^ and σ^–^ circular polarizations to probe emissions from the *K* and *K*′ valleys, respectively, as shown in Figure 2c,d. The comparable results for both polarizations indicate that the hysteresis is not valley-dependent. Additionally, a contrast can be observed in the hysteresis loop shape for the trion intensity between Figures 2b (bow-like) and 2d (butterfly like). This evolution occurred after subjecting the sample to lengthy and continuous voltage scans of Figure 2a,b and can be attributed to the passivation of specific activation channels for nonequilibrium electrons in the clinochlore layer. Furthermore, to examine the dependence of the memory effect on the clinochlore substrate, we conducted the same experiment on a reference sample. As shown in Figure S2, the reference sample exhibited a negligible hysteretic response. This observation aligns with previous reports of minor energy hysteresis for X^0^ emission in hBN-encapsulated 1L-MoS_2_ devices.^54^ Recently, devices utilizing 1L-MoSe_2_^53^ and 1L-MoS_2_^64^ atop a perovskite substrate demonstrated a significant hysteresis in the X^–^/X^0^ intensity ratio, which was attributed to a remanent polarization induced by the ferroelectric properties of the perovskite and underlines the fundamental role of the substrate in the hysteretic responses of such devices. We observe a similar hysteresis for the X^–^/X^0^ intensity ratio in the clinochlore sample (see Figure S4). However, here we focus on the individual memory effects for X^0^ or X^–^ emissions that are not reported in refs (53 and 64) and enable a more detailed comprehension of the distinct transient processes that govern these phenomena.^11^

**Figure 3:**
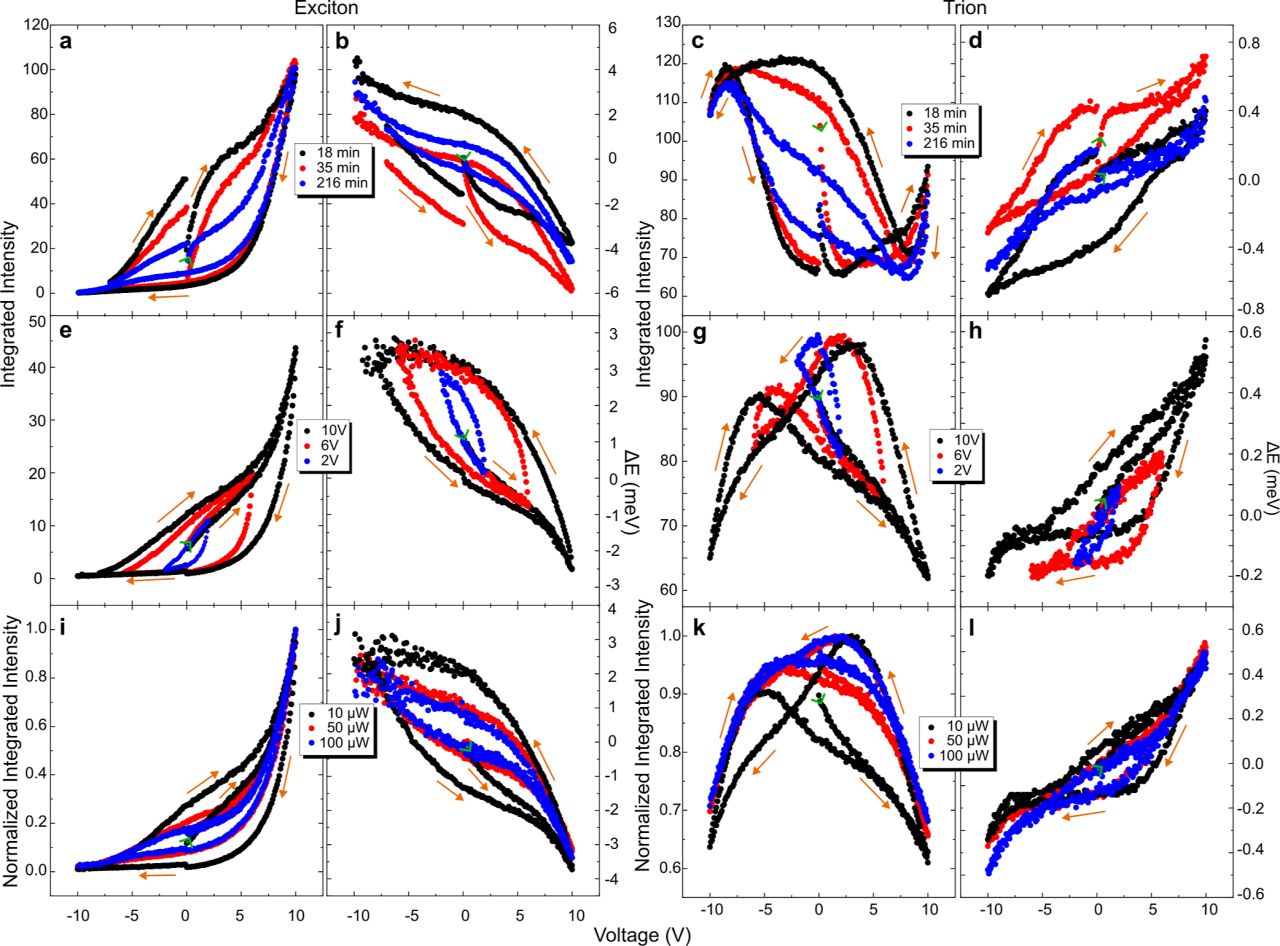
Hysteresis effect in the PL emission of the clinochlore device at different conditions of voltage sweep rate, voltage sweep amplitude, and excitation power. (a–d) Integrated intensity (a,c) and energy shift (b,d) of X^0^ (a,b) and X^–^ (c,d) emissions for different overall times of the gate voltage cycle before defect passivation. (e-h) Integrated intensity (e,g) and energy shift (f,h) of X^0^ (e,f) and X^–^ (g,h) emissions for different values of *V*_max_. (i–l) Normalized integrated intensity (i,k) and energy shift (j,l) of X^0^ (i,j) and X^–^ (k,l) emissions for different excitation powers. For all graphs, green arrows indicate where the measurement initiates and orange arrows denote the direction of the voltage sweep. The energy shift Δ*E* is relative to the first emission energy measured at 0 V. The voltage sweeps consist of a single cycle as follows: 0 V → *V*_max_ → – *V*_max_ → 0 V. The acquisition time for each PL spectrum was maintained at 0.2 s, with the overall time varied by adjusting the delay time between each spectrum.

To gain deeper insights into the hysteresis effect in the clinochlore device, we acquired PL spectra by sweeping the gate voltage in a single cycle (0 V → V_max_ → −V_max_ → 0 V) under various conditions: different sweep rates, sweep amplitudes, and excitation powers, as displayed in Figure 3. Figure 3a–d presents the intensity and energy of X^0^ and X^–^ emissions as a function of the gate voltage (*V*_max_ = 10 V), with measurements taken over periods of 18, 35, and 216 min. The results show that the hysteresis effect is more pronounced for shorter periods (black symbols) and that, according to ref (11), will be closer to the optimal memory response. Additionally, varying the sweep rate causes an energy shift in both the X^0^ and X^–^ emissions across all gate voltages. We observe a red shift (blue shift) for the X^0^ (X^–^) emission when increasing the cycle time from 18 to 35 min, with the energies for the 216 min measurement falling between those of the faster cycles. Figure 3e–h shows the hysteresis in intensity and energy of X^0^ and X^–^ emissions for *V*_max_ = 10 V (black), 6 V (red), and 2 V (blue) after defect passivation. Only the shape of the trion intensity fluctuation has been qualitatively modified. The hysteresis amplitude in both intensity and energy increases with higher *V*_max_. The excitation power dependence of the hysteresis effect is displayed in Figure 3i–l, showing the normalized intensity and energy of X^0^ and X^–^ emissions. The hysteresis magnitude is the largest at the lowest excitation power (10 μW) and exhibits similar reduced amplitudes at 50 and 100 μW.

To address the temperature dependence, we conducted additional measurements at 40 and 80 K. The results, presented in Figure S5 of the Supporting Information, demonstrate robust hysteresis loops at these higher temperatures, indicating that the memory effect persists across a range of temperatures.

**Figure 4:**
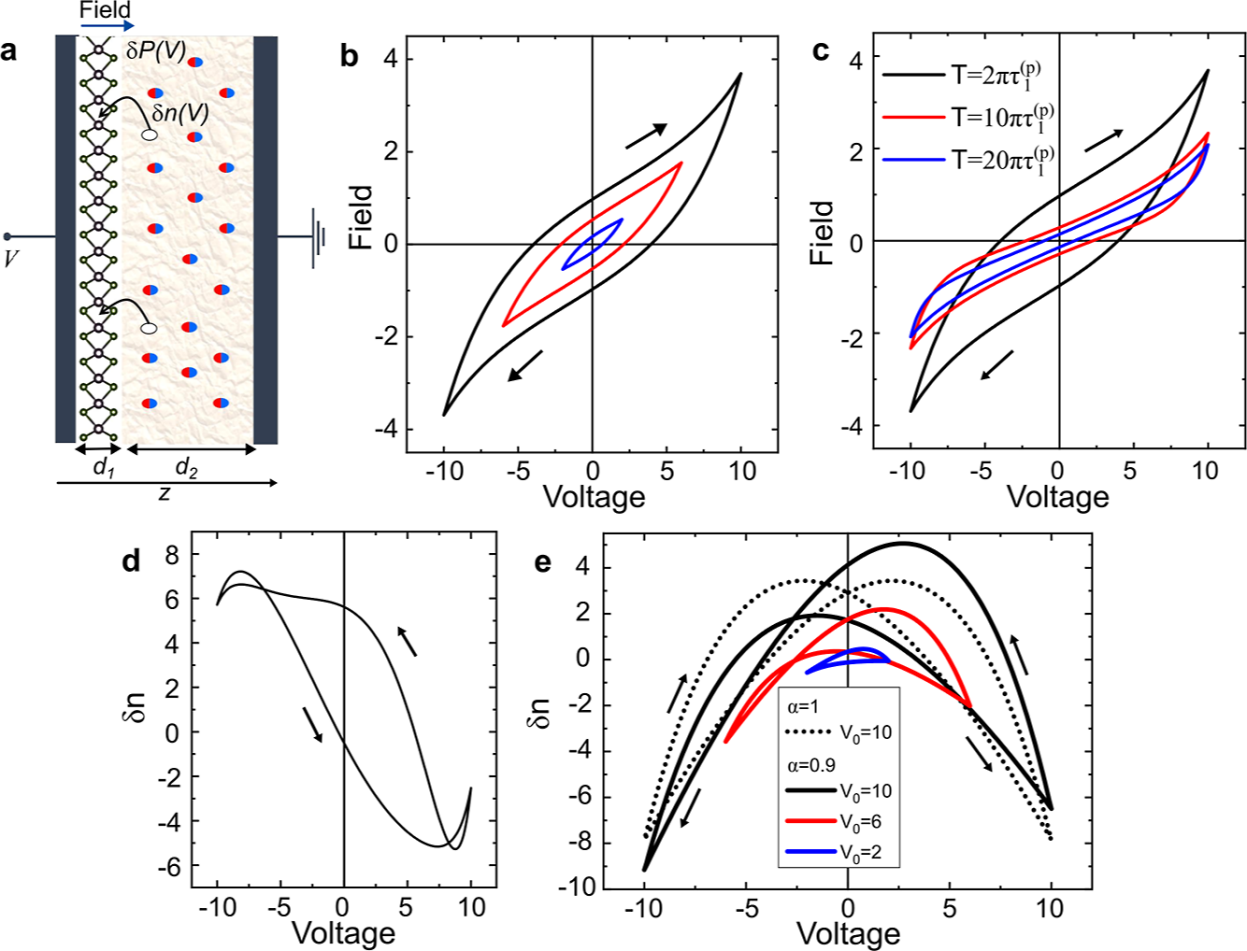
Electric hysteresis modeling of polarization and carrier population fluctuations in a 1L-TMD-based device. (a) Representation of a gated device composed by a 1L-TMD on a dielectric substrate. Polarization (δ*P*) and charge carrier (δ*N*) fluctuations occur in the substrate under an external electric field, impacting the optical properties of the TMD. Local field (*F*_TMD_) hysteresis considering polarization leakage channels and (b) increasing voltage amplitudes and (c) increasing voltage sweep periods. (d) Charge carrier fluctuation (δ*N*) hysteresis considering five trapping and release channels. (e) Charge carrier fluctuation hysteresis for different voltage amplitudes. The arrows in (b–e) denote the direction of the voltage sweep.

The topology of a hysteresis loop is shaped by concurrent processes involving the device’s internal state variables responding to external stimuli.^11^ These include fluctuations in the carrier population, ∑_*i*_δ*N*_*i*_, or polarization, ∑_*j*_δ*P*_*j*_, as described by eqs 1 and 2, respectively. Both processes are illustrated in Figure 4a. In the presence of carrier (dipoles) leakage,^11,65,66^ the electric field within the 1L-TMD, *F*_TMD_ ∝ ε_0_*V*/*d*_2_ + *∑*_*j*_δ*P*_*j*_(*t*), can be calculated to follow the clockwise hysteresis pattern shown in Figure 4b, with the loop size decreasing as the voltage amplitude is reduced. The transfer functions used for this calculation are shown in Figure S6a of the Supporting Information.

According to this model and Figure 2a,b, it is evident that the transition energies for excitons and trions vary proportionally to −*F*_TMD_ and *F*_TMD_, respectively. The exciton transition rate should also change proportionally to *F*_TMD_. Thus, the excitons contribute a transition rate-driven character to the Mem-emitter capabilities of the device. Consistent with the observed collapse of the memory response with an increasing sweep period, as shown in Figure 3a–d, the calculated hysteresis also shrinks in Figure 4c, reinforcing the existence of optimal driving conditions.

The trion emission, however, shows a distinct separation between energy shifts and intensity fluctuations both before and after defect passivation, as depicted in Figures 2 and 3. To reproduce the bow-like shape analogous to the observed experimental results in Figures 2b and 3c, obtained before defect passivation, Figure 4d illustrates the *∑*_*i*_δ*n*_*i*_ hysteresis calculated by combining five processes related to charge carrier population with contrasting relaxation times (assuming concomitant dynamics as detected in Figure 1e). Three of these processes are in the quasi-stationary regime (τ_*i*_^(*n*)^ ≪ *T*), while the other two have relaxation times comparable to those in the sweep period (τ_*i*_^(*n*)^ ∼ *T*). Their respective carrier transfer functions, *g*_*i*_^(*n*)^(*V*), are displayed in Figure S6b.

To reproduce the butterfly-like shape of the trion emission intensity after defect passivation, we consider two remaining dynamics with contrasting relaxation times and a slight inversion asymmetry, as shown in Figure 4e (see the passivated transfer functions in Figure S6c of the Supporting Information). The topology of this shape varies with the reduction of the voltage amplitude, analogous to the experimental response observed in Figure 3g. Thus, as anticipated in ref (11), trions can exhibit a hybrid response, providing population-driven Mem-emitter abilities while also displaying transition rate-driven features, similar to excitons. Hence, our modeled hysteresis loops suggest that for complex topologies multiple nonstationary fluctuations should be considered. Consequently, devices with a defect-rich dielectric substrate are more suited for a pronounced memory effect. This aligns with our experimental findings, where the clinochlore-based device, which contains significant Fe and H_2_O impurities,^49,51,52^ exhibits more complex and robust hysteresis compared to the hBN-based device.

While the observed optical memory effect in our clinochlore-based device highlights the potential of defect-rich substrates, similar phenomena have been reported for other materials, such as perovskite and ferroelectric substrates, which can modulate the PL properties of TMDs through mechanisms like remanent polarization.^53−56,67^ However, these previous studies primarily focused on phenomenological observations, whereas our work provides a deeper understanding by distinguishing transition rate- and population-driven contributions to the memory responses of exciton and trion emissions. Clinochlore offers distinct advantages as a nontraditional insulator, including its natural abundance, ease of exfoliation, and unique properties such as confined water nanochannels, which enable the exploration of dipole–water interactions under an external stimuli. These characteristics, coupled with their underexplored nature, open new avenues for investigating optical memory effects in TMD-based devices and other systems.

## 3 Conclusions

In summary, we present experimental evidence of a Mem-emitter device based on 1L-MoSe_2_ stacked on a few layers of clinochlore. We employed voltage-dependent PL experiments at low temperatures to investigate how this phyllosilicate affects the light emission of the TMD and to search for optical memory effects. Similar to the hBN-encapsulated 1L-MoSe_2_, the 1L-MoSe_2_/clinochlore device exhibited X^0^ and X^–^ emissions with narrow line widths, demonstrating its potential as an alternative 2D insulator for emitting vdWHS. Additionally, the 1L-MoSe_2_/clinochlore device showed a robust hysteresis in the intensity and energy of X^0^ and X^–^ MoSe_2_ emissions when an external electric field was applied, indicating a strong optical memory response. This contrasts with the weak hysteresis observed in the 1L-MoSe_2_/hBN reference device. The temporal dependence of the emission of the 1L-MoSe_2_/clinochlore device after a voltage pulse indicated distinct dynamic processes related to the internal state variables of the clinochlore substrate. These time-dependent processes govern the hysteresis of the optical observables. Therefore, the impurities in the clinochlore substrate introduce internal state variable processes, which are necessary for the device’s optical memory effect. Moreover, as the crystal shows good stiffness and flexibility in thin flakes, such an optical memory effect could also be fabricated over flexible or transparent substrates, as the device performance is independent of the support substrate. Consequently, our results pave the way for diverse memory functionalities based on TMD-based 2D vdWHS, highlighting the fundamental contribution of the clinochlore substrate to the reported Mem-emitter device.

## 4 Methods

### 4.1 Sample Preparation

Two sets of gated devices (clinochlore and reference samples) were fabricated following a fabrication process similar to that described in the sequence. All ultrathin flakes were obtained from the mechanical exfoliation of bulk crystals by applying the standard Scotch tape technique. The natural clinochlore crystal was extracted from the geological environment of Minas Gerais, Brazil and characterized by EDS using a Helios 5 PFIB CXe DualBeam microscope. The MoSe_2_ crystal was purchased from 2D semiconductors, while the hBN crystal was grown by the temperature-gradient method.^68^ The clinochlore (or hBN) crystal was mechanically exfoliated onto polydimethylsiloxane (PDMS) stamps and the selected flakes were dry transferred to prepatterned Ti/Au (5/35 nm) electrodes previously fabricated by nanofabrication techniques.^69^ The clinochlore (hBN) thickness selected for device fabrication was ∼26 (11) nm (measured by atomic force microscopy displayed in Figure S7) to avoid leakage current during the voltage bias application in the capacitor-like structure.^43^ To create the gated hBN/1L-MoSe_2_/clinochlore (hBN)/Au devices, we used the method described in ref (25) and a commercial transfer system from HQ graphene. The vdWH composed by hBN/1L-MoSe_2_/clinochlore (hBN)/Au were fabricated as follows: (i) we first picked the top hBN (∼10 nm) flake up from a SiO_2_/Si substrate with a polycarbonate (PC) membrane onto a PDMS stamp at 90 °C; (ii) a FLG flake was then picked up with the PC/hBN stamp at 60 °C; (iii) the PC/hBN/FLG stamp was aligned to the edge of the 1L-MoSe_2_ flake, and the PC/hBN/FLG/1L-MoSe_2_ was again picked up from the SiO_2_/Si substrate at 60 °C; and (iv) finally, PC/hBN/FLG/1L-MoSe_2_ was aligned to the clinochlore (or hBN) flake already transferred to the Ti/Au electrode and brought into contact at 180 °C, whereby PC adheres to the substrate, allowing PDMS to be peeled away, leaving PC/hBN/FLG/1L-MoSe_2_/clinochlore (hBN) on the Au electrode. PC is then dissolved in chloroform for ∼30 min at room temperature, leaving the hBN/FLG/1L-MoSe_2_/clinochlore (hBN) on the Au electrode. After the heterostructure assembly, an additional Ti/Au (5/50 nm) electrode was fabricated to contact the FLG flake and induce charge modulation to the MoSe_2_ layer by an external voltage bias in a capacitor-like design.

### 4.2 Optical Characterization

For the optical characterization, clinochlore and reference samples were placed in a helium closed cycle cryostat (Attocube/Attodry 1000) at 3.6 K and were electrically connected to a chip carrier for the application of the external voltage bias that was controlled by a source meter (Keithley 2400). The samples were excited by a linearly polarized laser beam with an excitation wavelength of 660 nm (Toptica—Ibeam). An aspheric lens (NA = 0.68) was used to focus the laser on the sample, and the backscattered PL signal was collected by the same lens and detected in a spectrometer equipped with a sensitive CCD camera (Andor/Shamrock—Idus). For the circular polarization measurements, the emitted signal was filtered with a linear polarizer and a quarter-wave plate.

## Supporting Information Available

Characterization of the insulating behavior of clinochlore flakes, characterization of the reference device, additional voltage sweeps in the clinochlore device, temperature dependence of hysteresis loops, transfer functions as a function of bias, and AFM characterization (PDF)
