# Supplementary material for: Optical Memory in a MoSe2/Clinochlore Device
Source: ACS Appl Mater Interfaces. 2025 Feb 13;17(8):12818–26. doi: 10.1021/acsami.4c19337 (PMC11874464; doi:10.1021/acsami.4c19337)
Supplement: Supplementary file 1 — am4c19337_si_001.pdf [file am4c19337_si_001.pdf]

Supporting Information for

Optical memory in a $MoSe_2$/Clinochlore device

Alessandra Ames[1], Frederico B. Sousa[1], Gabriel A. D. Souza[1], Raphaela de Oliveira[2], Igor R. F. Silva[1], Gabriel L. Rodrigues[3] Kenji Watanabe[4], Takashi Taniguch[5], Gilmar E. Marques[1], Ingrid D. Barcelos[2], Alisson R. Cadore[3], Victor López-Richard[1], and Marcio D. Teodoro[1]*

[1]Departamento de Física, Universidade Federal de São Carlos, 13565-905, São Carlos, São Paulo, Brazil

[2]Brazilian Synchrotron Light Laboratory (LNLS), Brazilian Center for Research in Energy and Materials (CNPEM), 13083-100, Campinas, São Paulo, Brazil

[3]Brazilian Nanotechnology National Laboratory (LNNano), Brazilian Center for Research in Energy and Materials (CNPEM), 13083-200, Campinas, São Paulo, Brazil

[4]Research Center for Electronic and Optical Materials, National Institute for Materials Science, 1-1 Namiki, Tsukuba 305-0044, Japan

[5]Research Center for Materials Nanoarchitectonics, National Institute for Materials Science, 1-1 Namiki, Tsukuba 305-0044, Japan

*Corresponding author: mdaldin@ufscar.br

This Supporting Information includes:

- Section S1. Insulating Behavior of Clinochlore Flakes
- Section S2. Characterization of the Reference Sample
- Section S3. Additional Voltage Sweeps in the Clinochlore Device
- Section S4. X^-/X^0 Intensity Ratio Hysteresis
- Section S5. Temperature dependence of the Hysteresis Loops

- Section S6. Transfer Functions

Section S1. Insulating Behavior of Clinochlore Flakes

An important property to be considered in our 1L-$MoSe_2$/clinochlore devices is the dielectric breakdown of clinochlore crystals. This electrical feature was investigated using parallel plane capacitors forming a gold(Au)/clinochlore/gold(Au) capacitor (see inset of Figure S1). Here we apply a potential difference through clinochlore crystals, with different thicknesses, and we measure the maximum potential before the dielectric breakdown (V_{BD}). Figure S1 brings three current *versus* voltage curves for different clinochlore flakes measured in forward and backward conditions. The curves demonstrate the high-insulating behavior of clinochlore crystals, independently of the direction of the electric field. Moreover, it shows that no hysteresis is observed in the bias loop (backward *versus* forward data). Therefore, these results indicate that we can neglect any significant charge tunneling in our 1L-$MoSe_2$/clinochlore device, once the V bias range applied in the PL experiments is much smaller than the V bias window expected to show significant charge transfer. Moreover, this observation also eliminates the possibility of a hysteresis effect induced only by the bias V.

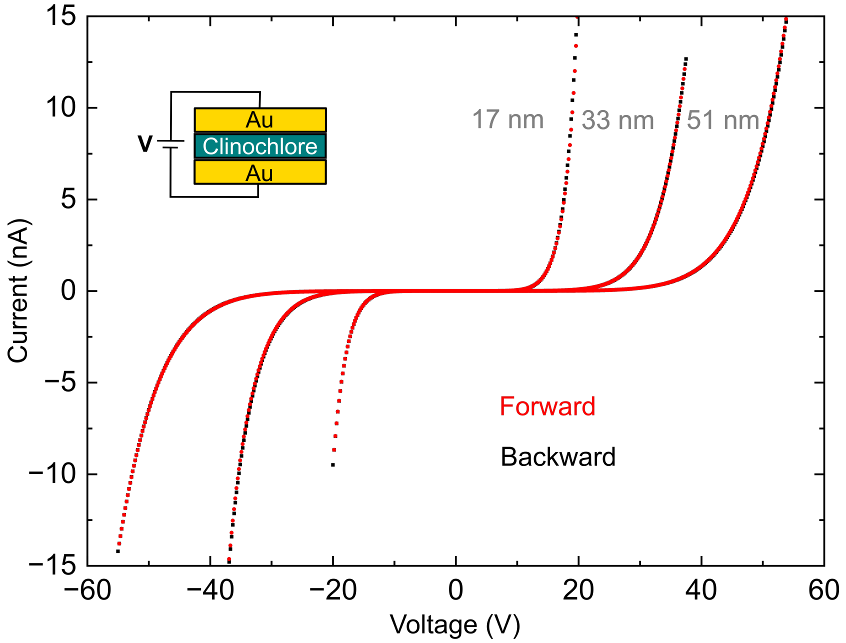

Figure S1: **Electrical breakdown of clinochlore flakes.** **a** Current versus Voltage bias at three representative clinochlore flakes with thicknesses of 17 nm, 33 nm, and 51 nm during forward (red) and backward (black) sweep.

Section S2. Characterization of the Reference Device

To gain deeper insights into the role of the clinochlore substrate in the optical memory effect reported in the manuscript, we also investigated a reference 1L-$MoSe_2$/hBN device. Figure S2a shows a schematic view of our reference device in a capacitor-like structure, in which the 1L-$MoSe_2$ is sandwiched between thin hBN flakes. Figure S2b presents the PL spectrum of the 1L-$MoSe_2$ obtained from the reference sample at 3.6 K and 0 V, exhibiting X^0 and X^- emissions. The X^0 (X^-) peak is centered at 1.644 eV (1.617 eV) and displays a FWHM of 4.3 meV (4.0 meV). We also acquired 1L-$MoSe_2$ PL spectra from the reference sample by sweeping the gate voltage in a single cycle (0 V $\rightarrow$ Vmax $\rightarrow$ $-$Vmax $\rightarrow$ 0 V). Figures S2c-f show the intensity and energy of X^0 and X^- emissions as a function of the gate voltage. A negligible hysteresis is observed for the reference device, corroborating the fundamental contribution of the clinochlore layers for the optical memory effect.

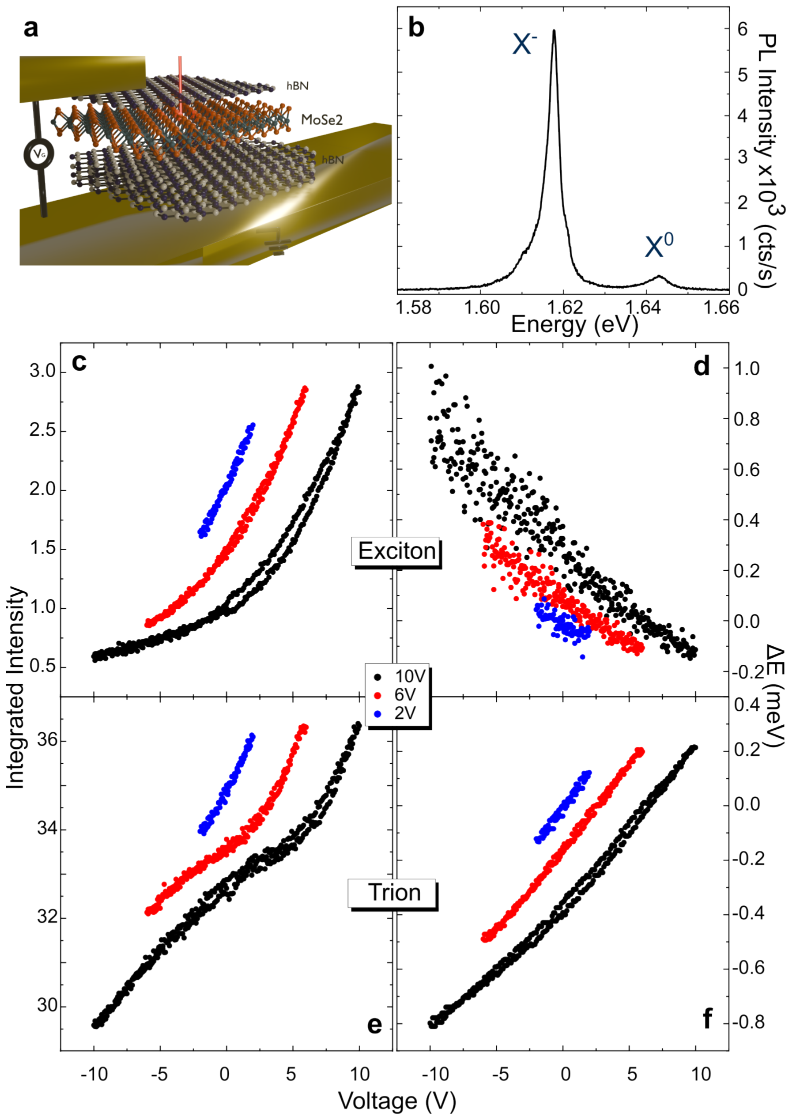

Figure S2: **Reference $MoSe_2$ device characterization.** **a** Schematic view of our hBN/1L-$MoSe_2$/hBN/Au-electrode device. **b** PL spectra of 1L-$MoSe_2$ acquired in the reference sample exhibiting X^0 and X^- emissions. **c-f** Integrated intensity (c,e) and energy shift (d,f) of X^0 (c,d) and X^- (e,f) emissions as a function of the gate voltage showing negligible hysteresis. The sweeps were performed for maximum voltages of 2 V (in blue), 6 V (in red), and 10 V (in black).

Section S3. Additional Voltage Sweeps in the Clinochlore Device

To check the reproducibility of the reported optical memory response, we obtained 1L-$MoSe_2$ PL spectra from the clinochlore sample by sweeping the gate voltage in two cycles with opposite directions. Figures S3a-d show the intensity and energy of X^0 and X^- emissions as a function of the gate voltage. The similar hysteresis observed in the closed loop part of the sweeps confirms the reproducible aspect of the optical memory effect.

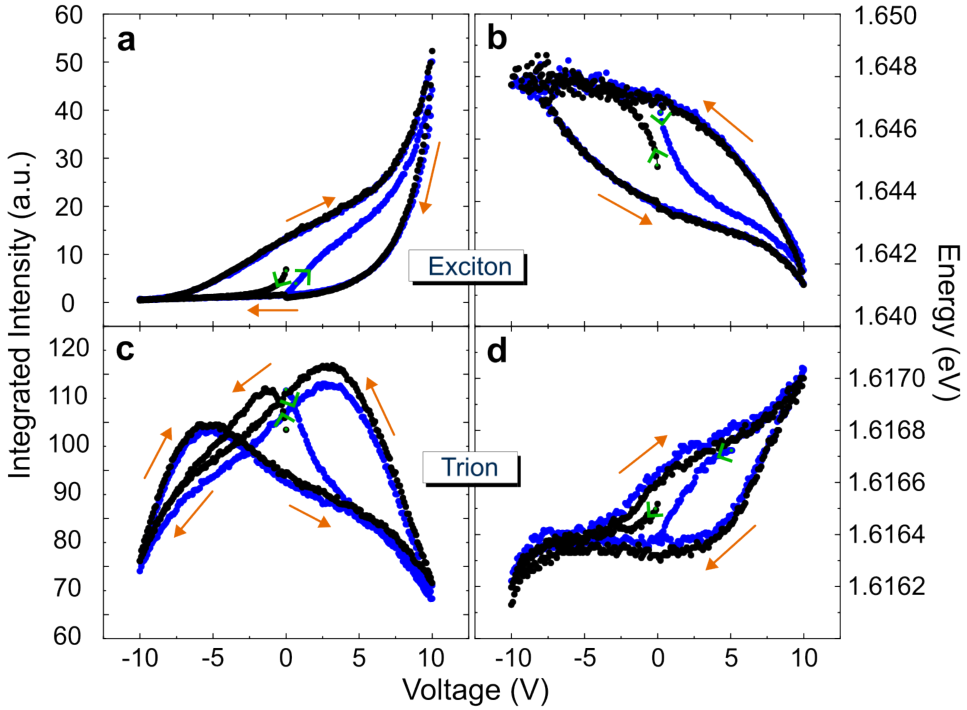

Figure S3: **Hysteresis effect in the PL emission of the clinochlore device for external voltage sweeps with opposite directions. a-d** Integrated intensity (a,c) and energy shift (b,d) of X^0 (a,b) and X^- (c,d) emissions as a function of the gate voltage. For each graph, two voltage sweeps ranging from -10 V to 10 V are displayed. The sweeps started at 0 V and were conducted in opposite directions. The green arrows indicate where the measurement initiates and the orange arrows denote the direction of the voltage sweep.

Section S4. X^-/X^0 Intensity Ratio Hysteresis

As mentioned in the manuscript, hysteresis in the X^-/X^0 intensity ratio were recently reported for devices based on 1L-$MoSe_2$[1] and 1L-MoS_2[2] atop a perovskite substrate due to a remanent polarization. Similarly, Figure S4 presents a robust hysteresis for the intensity ratio between X^- and X^0 1L-$MoSe_2$ emissions obtained from the 1L-$MoSe_2$/clinochlore device. However, while the hysteresis attributed to a remanent polarization generally exhibit a slower variation of the observables by changing the voltage sweep direction[1,2], here we observe an abrupt modification in the X^-/X^0 intensity ratio when the sweep direction is altered. This indicates that the dynamical processes that govern the optical memory effect of the clinochlore device are more complex than a simple remanent polarization.

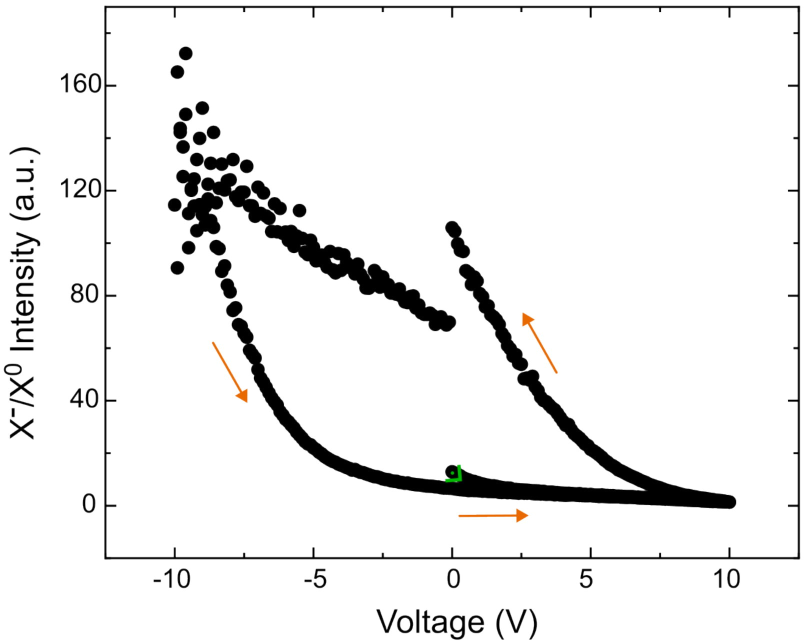

Figure S4: **X^-/X^0 intensity ratio hysteresis of the clinochlore device. a,b** Intensity ratio between X^- and X^0 emissions as a function of the gate voltage. The green arrow indicates where the measurement initiates and the orange arrows denote the direction of the voltage sweep.

Section S5. Temperature dependence of the Hysteresis Loops

The hystereses loops were also mensured at 40 K and 80 K. In the Figure S5, the red data represent the energy shift of the photoluminescence (PL) peaks—exciton on the left and trion on the right—while the black data correspond to the intensity of these peaks. The results clearly demonstrate that the memory effect persists across this temperature range, confirming the robustness and reproducibility of the observed hysteresis loops.

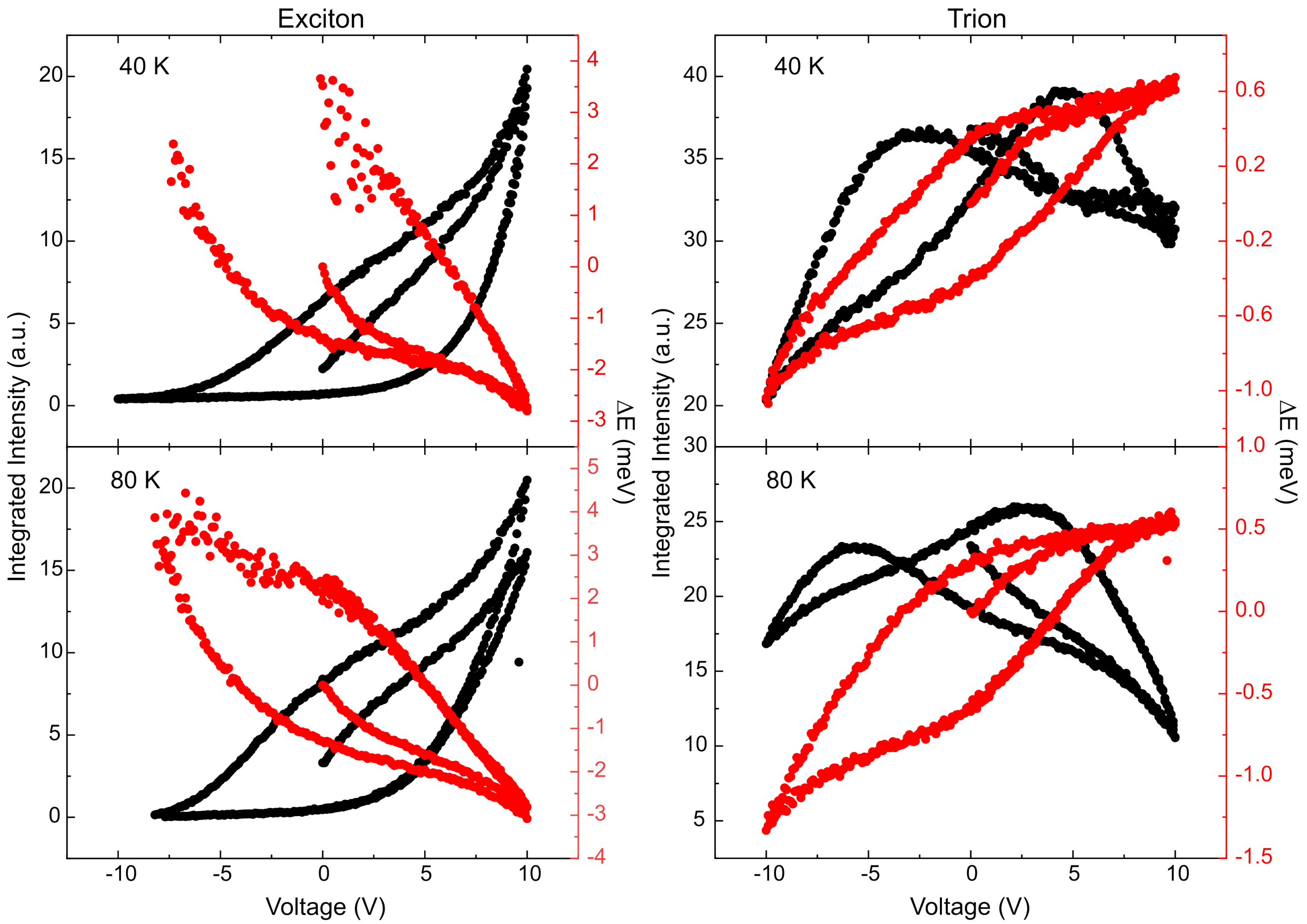

Figure S5: PL Integrated Intensity and energy variation for exciton (left) and trion (right) at 40 K and 80 K.

Section S6. Transfer Functions

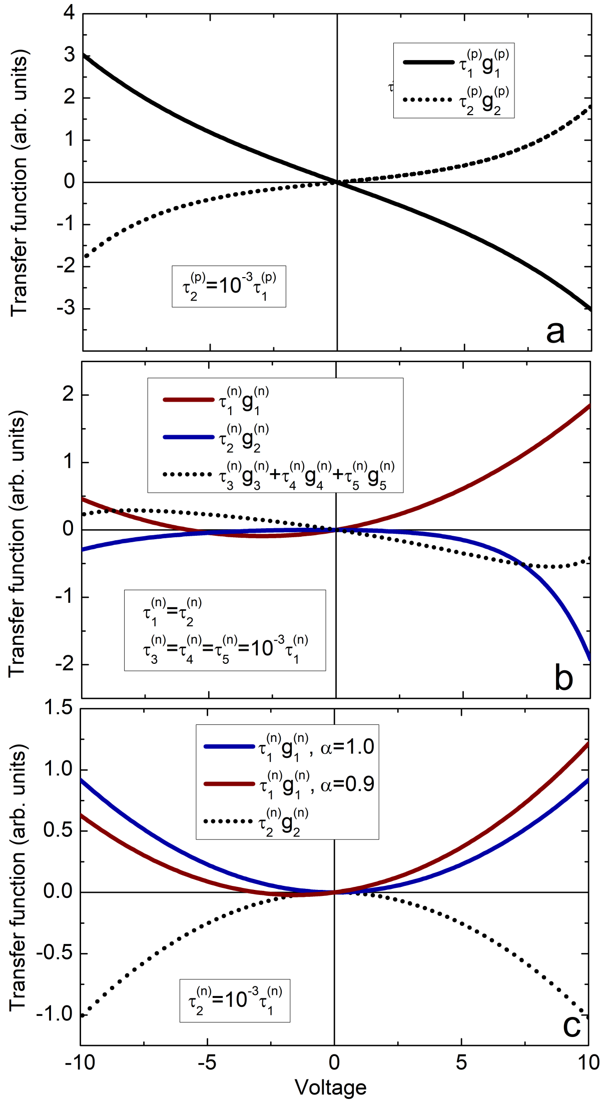

Figure S6: **Non-equilibrium transfer functions a** Nonequilibrium polarization transfer functions for a leaking mechanism (solid curve) and a nonlinear polarization fluctuation (doted curve) used for the local electric field calculation displayed in panels 4 b and c of the manuscript. **b** Nonequilibrium charge transfer functions from the substrate to the TMD monolayer as function of applied voltage used in panel 4 d of the manuscript. **c** Nonequilibrium charge transfer functions from the substrate to the TMD monolayer as function of applied voltage used in panel 4 e of the manuscript.

Each independent mechanism j contributing to the non-equilibrium polarization fluc-

tuation is characterized by its relaxation time $\tau_j^{(p)}$ and the polarization transfer function $g_j^{(p)}(V)$, given by

$$g_j^{(p)} = \pm \frac{e\lambda_j}{2\eta} \left[\exp\left(\eta \frac{eV}{k_B Teff} \right) - \exp\left(-\eta \frac{eV}{k_B T_{eff}} \right) \right], \tag{S1}$$

where e is the electron charge, $\lambda_j = \dfrac{4\pi m^* (k_B T_{eff})^2 \exp\left(-\frac{E_j^b}{k_B T_{eff}} \right)}{(2\pi\hbar)^3}$, T_{eff} is the effective temperature, E_j^b is the activation barrier for each non-equilibrium process, and $\eta < 1$ represents the local voltage efficiency drop. The positive sign in Eq. S1 corresponds to the contribution from charge bouncing within localization sites in the substrate, while the negative sign corresponds to the contribution from leakage [3,4]. The functions used to generate the results shown in Figures 4b and 4c of the manuscript are presented in Figure S3a.

The transfer or generation rate for charge fluctuations with relaxation time $\tau_j^{(n)}$ can be expressed as

$$g_j^{(n)} = \pm \frac{\lambda_j A}{\eta} \left[\exp\left(\mp \eta_L \frac{eV}{k_B T} \right) + \exp\left(\pm \eta_R \frac{eV}{k_B T} \right) - 2 \right], \tag{S2}$$

where A is the device area, and $\eta_R = \dfrac{\eta}{1+\alpha}$ and $\eta_L = \dfrac{\eta\alpha}{1+\alpha}$, with $\alpha \equiv \eta_L/\eta_R \in [0, \infty)$ quantifying the local symmetry break. The case of perfect symmetry, $\alpha = 1$, corresponds to $\eta_R = \eta_L = \eta/2$ [5].

The transfer functions used to obtain the results in Figure 4c are presented in Figure S3b, while the corresponding transfer functions used for Figure 4d are shown in Figure S3c.

Section S7. AFM characterization

The device's topology was also analyzed to gain a better understanding of the layer thicknesses. Figure S7(a) highlights the optical image of the device, indicating the regions presented in Figures S7(b) and S7(c). The main components of the device are labeled in Figure S7(b), while the thicknesses of hBN and clinochlore were measured along the dotted line shown in Figure S7(c). The resulting topographic profile is displayed in Figure S7(d).

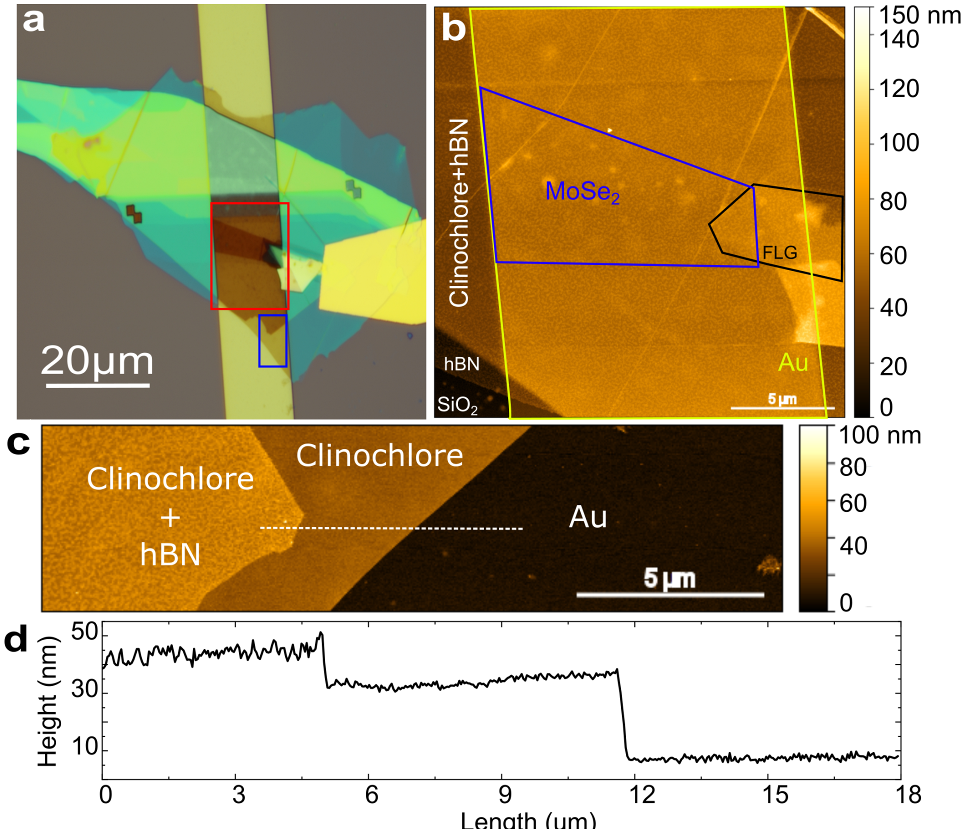

Figure S7: **a** Optical image with AFM region highlighted, **b** AFM image, **c** AFM region of **d** the topography profile of the device.

[1] J. Choi, K. J. Crust, L. Li, K. Lee, J. Luo, J.-P. So, K. Watanabe, T. Taniguchi, H. Y. Hwang, K. F. Mak, J. Shan, and G. D. Fuchs, "Tuning Exciton Emission via Ferroelectric Polarization at a Heterogeneous Interface between a Monolayer Transition Metal Dichalcogenide and a Perovskite Oxide Membrane," *Nano Letters*, jul 2024.

[2] T. Pucher, S. Puebla, V. Zamora, E. Sánchez Viso, V. Rouco, C. Leon, M. Garcia-Hernandez, J. Santamaria, C. Munuera, and A. Castellanos-Gomez, "Strong Electrostatic Control of Excitonic Features in MoS2 by a Free-Standing Ultrahigh-κ Ferroelectric Perovskite," *Advanced Functional Materials*, vol. n/a, p. 2409447, aug 2024.

[3] X. Wei, Y. Feng, L. Hang, S. Xia, L. Jin, and X. Yao, "Abnormal c–v curve and clockwise hysteresis loop in ferroelectric barium stannate titanate ceramics," *Materials Science and Engineering: B*, vol. 120, no. 1, pp. 64–67, 2005. The 8th International Symposium on Ferroic Domains (ISFD-8, 2004).

[4] D.-G. Jin, S.-G. Kim, H. Jeon, E.-J. Park, S.-H. Kim, J.-Y. Kim, and H.-Y. Yu, "Improvement of polarization switching in ferroelectric transistor by interface trap reduction for brain-inspired artificial synapses," *Materials Today Nano*, vol. 22, p. 100320, 2023.

[5] V. Lopez-Richard, R. S. W. Silva, O. Lipan, and F. Hartmann, "Tuning the conductance topology in solids," *Journal of Applied Physics*, vol. 133, no. 13, p. 134901, 2023.
